# Tracking the History and Ecological Changes of Rising Double-Crested Cormorant Populations Using Pond Sediments from Islands in Eastern Lake Ontario

**DOI:** 10.1371/journal.pone.0134167

**Published:** 2015-07-27

**Authors:** Emily M. Stewart, Neal Michelutti, Sarah Shenstone-Harris, Christopher Grooms, Chip Weseloh, Linda E. Kimpe, Jules M. Blais, John P. Smol

**Affiliations:** 1 Paleoecological Environmental Assessment and Research Laboratory, Department of Biology, Queen’s University, Kingston, Ontario, Canada, K7L 3N6; 2 Canadian Wildlife Service, Environment Canada, Toronto, Ontario, Canada, M3H 5T4; 3 Department of Biology, University of Ottawa, Ottawa, Ontario, Canada, K1N 6N5; University of Regina, CANADA

## Abstract

In the Laurentian Great Lakes region, the double-crested cormorant (*Phalacrocorax auritus*) has seen a thousand-fold population increase in recent decades. These large colonies of birds now often conflict with socioeconomic interests, particularly due to perceived competition with fisheries and the destruction of terrestrial vegetation in nesting habitats. Here we use dated sediment cores from ponds on islands in eastern Lake Ontario that receive waste inputs from dense colonies of cormorants and ring-billed gulls (*Larus delawarensis*) to chronicle the population rise of these species and assess their long-term ecological impacts. Modern water chemistry sampling from these sites reveals drastically elevated nutrient and major ion concentrations compared to reference ponds not influenced by waterbirds. Geochemical tracers in dated sediment cores, particularly δ^15^N and chlorophyll-*a* concentrations, track waterbird influences over time. Fossil diatom assemblages were dominated by species tolerant of hyper-eutrophic and polluted systems, which is in marked contrast to assemblages in reference sites. In addition to establishing long-term ecological impacts, this multi-proxy paleoecological approach can be used to determine whether islands of concern have been long-term nesting sites or were only recently colonized by cormorant or ring-billed gull populations across the Great Lakes, facilitating informed management decisions about controversial culling programs.

## Introduction

Animals that deposit large amounts of waste products to specific receptor sites are gaining increased attention due to their ability to shape the structure and function of ecosystems [1]. In addition to humans, prominent examples include migrating salmon [2] and Arctic seabirds [3] that may potentially focus large quantities of nutrients and bioaccumulated contaminants into their spawning and nesting areas, respectively. Temperate seabirds, such as gulls and cormorants, have also been assessed for their role in transporting marine-derived nutrients to nesting islands in the Gulf of Maine [4]. A topical example from the freshwater realm is that of the double-crested cormorants (*Phalacrocorax auritus*; hereafter cormorants), whose exponential population rise in North America has raised concern over their ecological impacts, many of which have socioeconomic implications. Cormorants have been closely intertwined with human activities for centuries and are now controversial in the North American Great Lakes region because large nesting colonies concentrate toxic amounts of ammonia-rich guano that kill native vegetation on islands, including portions of what is left of the northernmost stands of Carolinian forests not already impacted by human development [5]. In addition, cormorants are thought to interact with fish farms along the Mississippi River in the winter [6] and have been possibly linked to declining sports fisheries (i.e. smallmouth bass, *Micropterus dolomieu*) in the Great Lakes [7]. Cormorants have also been cited as a potential threat to the nesting habitats of other bird species due to their aggressive competition for space and resources [8]. The anti-cormorant sentiment may be undeserved, however, as studies show that cormorants eat mainly small non-sport fish and do not necessarily overlap in feeding niche with sport fishes [9]. Nonetheless, it is clear that cormorant numbers in the Great Lakes region of North America have increased exponentially and their potential ecological impacts warrant investigation.

In the mid-20^th^ century, cormorants in the Great Lakes were nearly extirpated due to the heavy use of organochlorine pesticides (especially DDT) that caused reproductive failure, as well as from human eradication attempts due to their perceived role as competitors with commercial fisheries [10, 11]. Ironically, after their near extirpation, cormorants became a “poster-species” for environmental recovery in the Great Lakes region. Following the ban of DDT in 1972, their numbers began to recover, also aided in part by increases in the invasive alewife (*Alosa pseudoharengus*), which was then the main component of their summer diets [12]. Cormorants in the Great Lakes have since experienced exponential population growth beyond historical levels of the pre-DDT era, moving from a low of ~136 breeding pairs in the 1970s to over 100,000 breeding pairs in the early 2000s [13]. Though numbers of breeding cormorants on the Great Lakes have been characterized recently [10], data are absent for population numbers prior to the early-1900s, and data are sparse for even before the mid-1900s, as large scale inventories of waterbirds did not begin until 1976 [14]. Here, we employ a limnological and paleoecological approach to determine the effects of water bird populations on island pond ecology and to determine if the recent increase in cormorants is unprecedented.

Lake Ontario contains many islands, some of which have notable breeding and post-breeding populations of cormorants and other waterbirds. In terms of the entire Great Lakes system, Lake Ontario currently hosts the highest concentrations of cormorant nests, and eastern Lake Ontario (our study location) has numerous islands that continue into the St. Lawrence River and provide ideal cormorant nesting habitat [10]. Large, densely-packed cormorant colonies release tremendous amounts of nutrient-rich wastes to the surrounding environment, including guano, regurgitated food, feathers, and carcasses. Small freshwater ponds that exist on these nesting islands act as receptor sites for waterbird wastes. The nesting islands also host dense colonies of ring-billed gulls (*Larus delawarensis*), which are by far the most common waterbird on the Great Lakes and have also had population increases beginning in the early 20^th^ century [15, 16].

Here, we track the rise in cormorants and ring-billed gulls, as well as their ecological impacts, using modern limnological analyses coupled with a multi-proxy paleolimnological approach. We reconstruct changes in bird influence and aquatic production over the past ~150 years using stable nitrogen isotopes (δ^15^N), spectrally-inferred chlorophyll-*a*, and fossil diatom assemblages. Specifically, we demonstrate how this approach can be used to assess the impacts of waterbirds on water quality and obtain data on past population histories that are critical to making informed wildlife management decisions.

## Site Description

**Fig 1:**
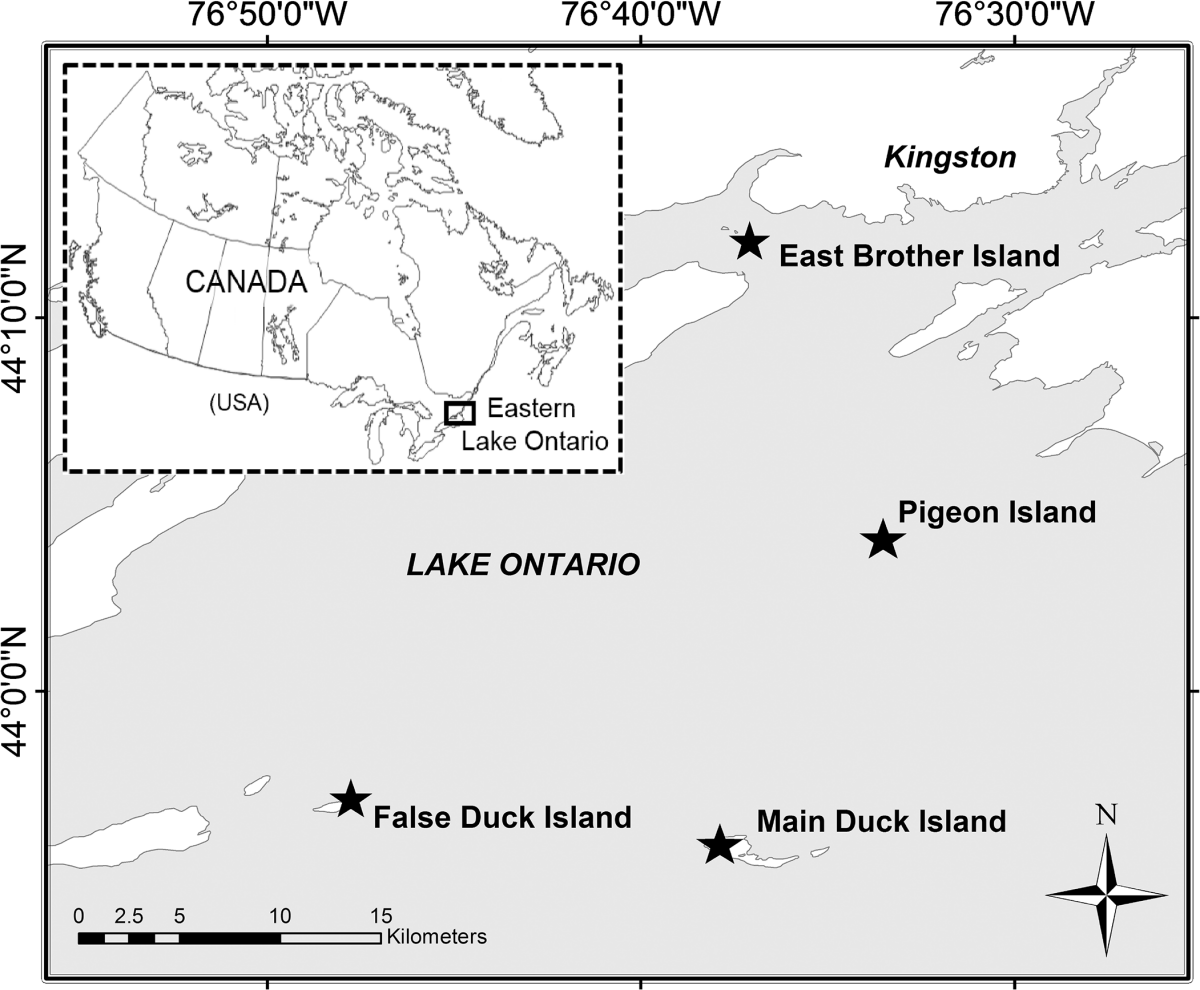
A map of eastern Lake Ontario near Kingston, ON. Study ponds (with name and impact level) are on East Brother Island (EB, high-impact), Pigeon Island (PGN, high-impact), False Duck Island (FD1, low-impact), and Main Duck Island (MD2, no-impact).

Small freshwater ponds from four islands in the Canadian portion of eastern Lake Ontario were sampled for modern water chemistry and for sediment cores. Ponds from two of these islands—East Brother Island (pond EB) (44°12’18.43”N, 76°37’28.33”W) and Pigeon Island (pond PGN) (44°03’59.41”N, 76°32’51.51”W) (Fig 1)–were considered “high-impact” sites as they were surrounded by large numbers of cormorants and ring-billed gulls. Permission to sample EB was granted by private landowner, John Weatherall, and by the Canadian Wildlife Service for PGN. Cormorant occupation was first noted and measured on East Brother Island in 2001 with ~200 nests (2 individuals per nest), and with an average of ~1300 nests between 2003 and 2012 (S1 Table). Cormorants were first noted on Pigeon Island in 1962 with a few reproductively unsuccessful cormorant pairs [17]. During a 1989–1991 census, ~600 cormorant pairs were noted breeding on Pigeon Island [17]. However, Pigeon Island was dominated by a large colony of ring-billed gulls that had ~5000 nests in 1990 [14], but later deserted the island sometime between 1991 and 1997 [17]. Pigeon Island now hosts a colony of ~2100 cormorants (Table 1). The high-impact ponds on East Brother and Pigeon islands have virtually no remaining terrestrial vegetation in their catchment, except dead trees and a few shrubs and vines that can tolerate the high levels of ammonia from the excessive guano deposition. Cormorant breeding pairs arrive on these islands in April, breed until early August, and stay until early November [10, 17]. Guano, feathers, egg shells, regurgitated fish, and some cormorant carcasses were commonly noted during the breeding season in ponds EB and PGN.

**Table 1:** Cormorant impact and morphological characteristics of study ponds.

Pond ID	EB	PGN	FD1	MD2
Impact	High	High	Low	None
Cormorant arrival	2001	1962	1986	-
Approx. # nests	1300	2100	>10	0
Diameter (m)	~50	~20	~20	~100
Max. depth (m)	~0.5	~0.1	~0.45	~1
pH	7.6	7.0	7.3	7.6
Specific conductivity (μS/cm)	800	1650	433	252
Total unfiltered phosphorus (μg/L)	3500	-	542	86.9
Total unfiltered nitrogen (mg/L)	8.79	-	7.5	1.46

Pond abbreviations are as follows: East Brother (EB), Pigeon (PGN), False Duck Pond 1 (FD1), and Main Duck Pond 2 (MD2). The arrival of cormorants to East Brother and False Duck is based on data from D.V.C. Weseloh (S1 Table) and based on data from [17] for PGN. Numbers for nests, as well as pond diameter and depth, are estimates made in the field at the time of sampling. Details of water chemistry sampling methods and dates are provided in the materials and methods section.

In addition to high-impact ponds, we sampled two reference ponds on southwestern False Duck Island (pond FD1) (43°56’53.98”N, 76°48’11.94”W) and Main Duck Island (pond MD2) (43°55’28.39”N, 76°36’51.34”W), both of which have little-to-no cormorant or gull influence and are part of marshes (Fig 1). A research permit for collections on False Duck Island was granted by the Canadian Wildlife Service. A research and collection permit was issued by the Parks Canada Agency for our sampling on Main Duck Island. The study pond on False Duck Island has evidence of some modest cormorant activity in its catchment and thus we classified it as a “low-impact” site. Cormorants were first noted on False Duck Island in 1986 with ~100 nests (S1 Table). The pond on Main Duck Island has no evidence of nesting cormorants in its catchment and so we classified it as a “no-impact” site. Recent approximate cormorant numbers on all study islands, as well as pond morphology and water chemistry, are summarized in Table 1.

## Materials and Methods

### Water chemistry

Water samples were taken from EB (high-impact) and FD1 (low-impact) on May 28^th^ 2012, as well as from MD2 (no-impact) on September 11^th^ 2013, and were sent to the National Laboratory for Environmental Testing (Burlington, ON) to be analyzed for nutrients, major ions, and trace metals using their standard procedures [18, 19]. Specific conductivity and pH measurements were taken on-site using a calibrated YSI meter for all aforementioned ponds, as well as from PGN (high-impact) on June 16^th^ 2011 (which had very little water at the time). Additional water chemistry was acquired using the same methods for the high-impact pond (EB) twice in 2013 (S2 Table). Seasonal differences (either environmental or bird-related) were assumed to be negligible between measurements for high-impact ponds collected at the end of May and middle of June. Measurements from the no-impact pond (MD2) were taken closer to the end of the season, and so some seasonal differences (due to evaporation) may be apparent.

### Sediment sampling and dating

A sediment core was retrieved from each pond using a Glew [20] corer with a 7.6-cm diameter Lexan tube on the following dates: EB on May 28^th^ 2012, PGN and FD1 on June 16^th^ 2011, and MD2 on September 11^th^ 2013. Each core was sectioned into 0.5-cm intervals using a Glew [21] extruder. Freeze-dried sediments from all sites were dated using excess ^210^Pb activities, and independently verified with ^137^Cs. All radioisotopes were measured at the Paleoecological Environmental Assessment and Research Laboratory (PEARL) (Queen’s University, Kingston, ON) using an Ortec high purity Germanium gamma spectrometer (Oak Ridge, TN, USA). Chronologies based on ^210^Pb data were developed using the constant rate of supply (CRS) model [22] at PEARL with the ScienTissiME (Barry’s Bay, ON, Canada) software created for Matlab. Only the ^137^Cs independent dating marker defining the ~1963 peak in atomic bomb testing was used for EB, as we did not attain reliable ^210^Pb dates (S1 Fig).

### Sedimentary δ^15^N and spectrally-inferred chlorophyll-a

Sediments from every other 0.5-cm interval for all ponds were analyzed for δ^15^N at the University of Ottawa (Ottawa, ON, Canada) following procedures modified from Yamamuro and Kayanne [23]. Briefly, (unacidified) freeze-dried sediments were combusted at 1800°C in an elemental analyzer (EA 1110, CE Instruments, Italy), and resultant gases were run through an isotope ratio mass spectrometer (Delta-Plus Advantage IRMS, ThermoFinnigan, Germany) using a Conflo III Interface. Internal standards calibrated with international standards (IAEA-CH-6, IAEA-NBS22, IAEA-N1, IAEA-N2, USGS-40, USGS-41, with a precision of ±0.2‰) were used to normalize data. Elevated sedimentary δ^15^N in the study ponds would indicate the influence of birds, as cormorants in eastern Lake Ontario have δ^15^N values of ~15‰ because they occupy the top of the pelagic food web [24]. In contrast, ring-billed gulls have a more varied diet and often feed lower on the food chain (i.e. on landfills [25]) compared to cormorants, with δ^15^N values for various tissues ranging from 7–11‰ [26]. For reference, pre-industrial sediments of Lake Ontario prior to any large-scale human activities had a background δ^15^N value of ~4‰ [27].

Sedimentary chlorophyll-*a* concentrations were used to determine if coincident increases in primary production occurred with the addition of cormorant wastes, as tracked by δ^15^N. Chlorophyll-*a* concentrations in freeze-dried and sieved (using a 125-μm mesh) sediments were determined using visible near-infrared reflectance spectroscopy, according to the methods outlined in [28]. Every 0.5-cm interval was analyzed for EB, PGN, and MD2. Every other interval was analyzed for FD1. Importantly, this approach tracks both the primary chlorophyll-*a* pigment, plus all chlorophyll-*a* isomers and degradation products, including pheophytin *a* and pheophorbide *a*, (hereafter collectively referred to as “chl-*a*”).

### Subfossil diatom assemblages

Subfossil diatom assemblages were isolated from the sediments (of every other interval) by digesting 0.05-g subsamples of freeze-dried sediment in a 1:1 (molar weight) mixture of H_2_SO_4_ and HNO_3_ at 80°C for 2 hours, and then aspirating samples to neutral pH before mounting dilutions permanently on slides using Naphrax [29]. Diatom valves were identified with a Leica DMR HC light microscope at 1000x magnification, following the taxonomy of Krammer and Lange-Bertalot [30]. A minimum of 200 diatom valves was identified for each interval and the chrysophyte cyst to diatom ratio (C:D) was also calculated for each sediment section [31]. The C:D can be used as an indicator of trophic status in temperate freshwaters, as chrysophytes are typically more common in oligotrophic waters [31]. As diatoms are sensitive indicators of many limnological variables (e.g. pH, nutrients) [32], they can be used to track environmental changes in our ponds that may then be related to cormorant occupation by our geochemical analysis.

## Results

### Water chemistry

**Fig 2:**
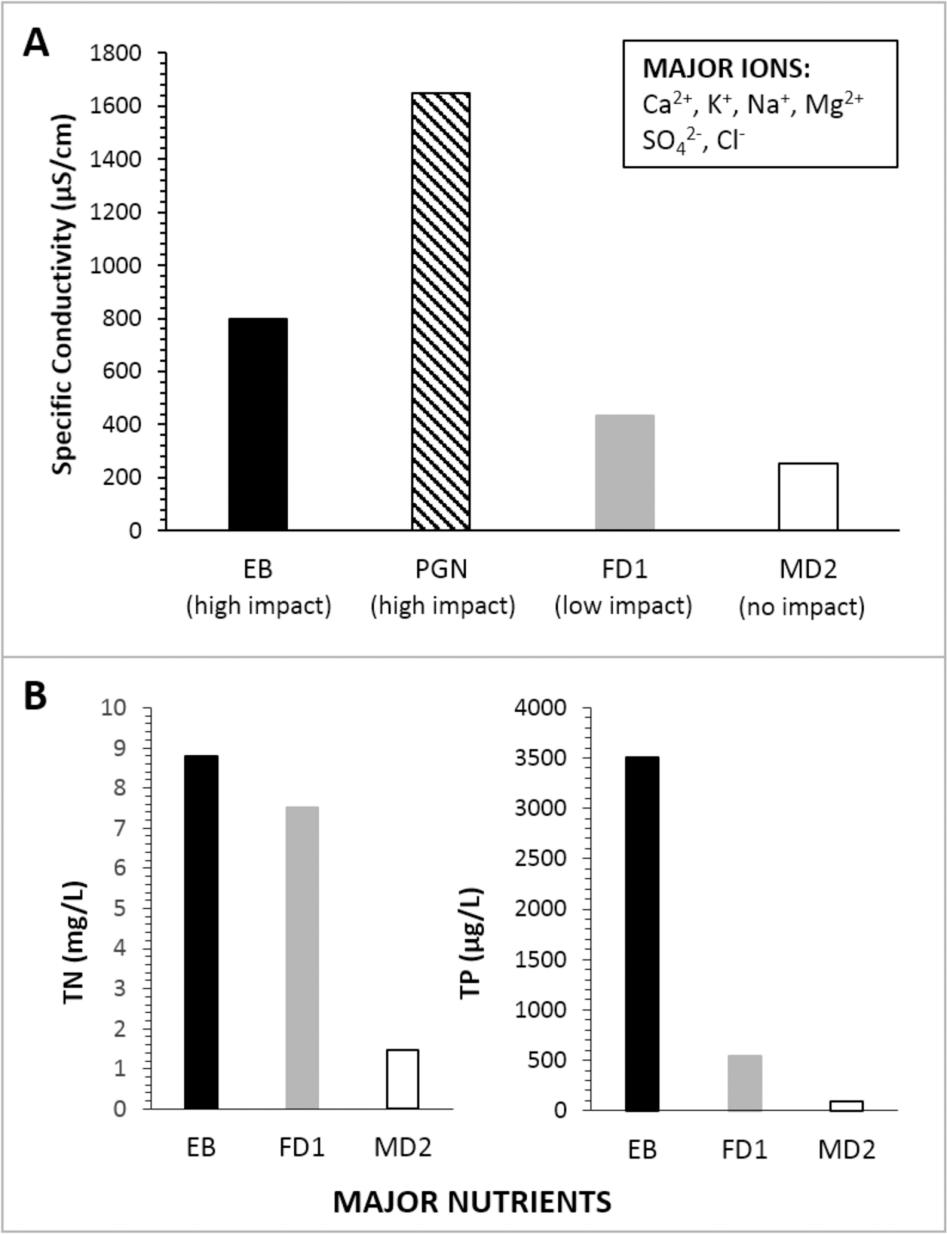
Water chemistry for impacted and reference sites. (A) Specific conductivity (μS/cm) in high-impact ponds in black and hashed (ponds EB and PGN, respectively), the low-impact pond (FD1) in grey, and the no-impact pond (MD2) in white. Major cations and anions are listed in the inset by decreasing concentration. (B) Major nutrients in three study ponds, including total unfiltered nitrogen (TN) concentrations (mg/L) and total unfiltered phosphorus (TP) concentrations (μg/L).

Large differences in water chemistry were noted between the high-impact pond (EB) and the reference ponds (FD1 and MD2), with particularly elevated concentrations of ions (reflected in specific conductivity) and nutrients in the pond heavily influenced by cormorant waste inputs (Table 1, Fig 2). Major ions (K^+^, Mg^2+^, Na^+^, Ca^2+^, Cl^-^, SO_4_^2-^) were enriched in the high-impact pond (EB) by 5–110 times, with the greatest enrichment occurring in potassium ions (S3 Table). The specific conductivity increased by a factor of two between each pond moving along a gradient of increasing cormorant influence (i.e. MD2 to FD1 to EB to PGN) (Fig 2A). Total unfiltered phosphorus (TP-u) was 3500 μg/L in EB, and was 6 times lower (but still high) in FD1 (542 μg/L) and 40 times lower in MD2 (86.8 μg/L) (Fig 2B). Total unfiltered nitrogen (TN-u) was slightly higher in EB (8.79 mg/L) than in FD1 (7.5 mg/L), and much lower in MD2 (1.46 mg/L) (Fig 2B).

### Radiometric dating of sediment cores

**Fig 3:**
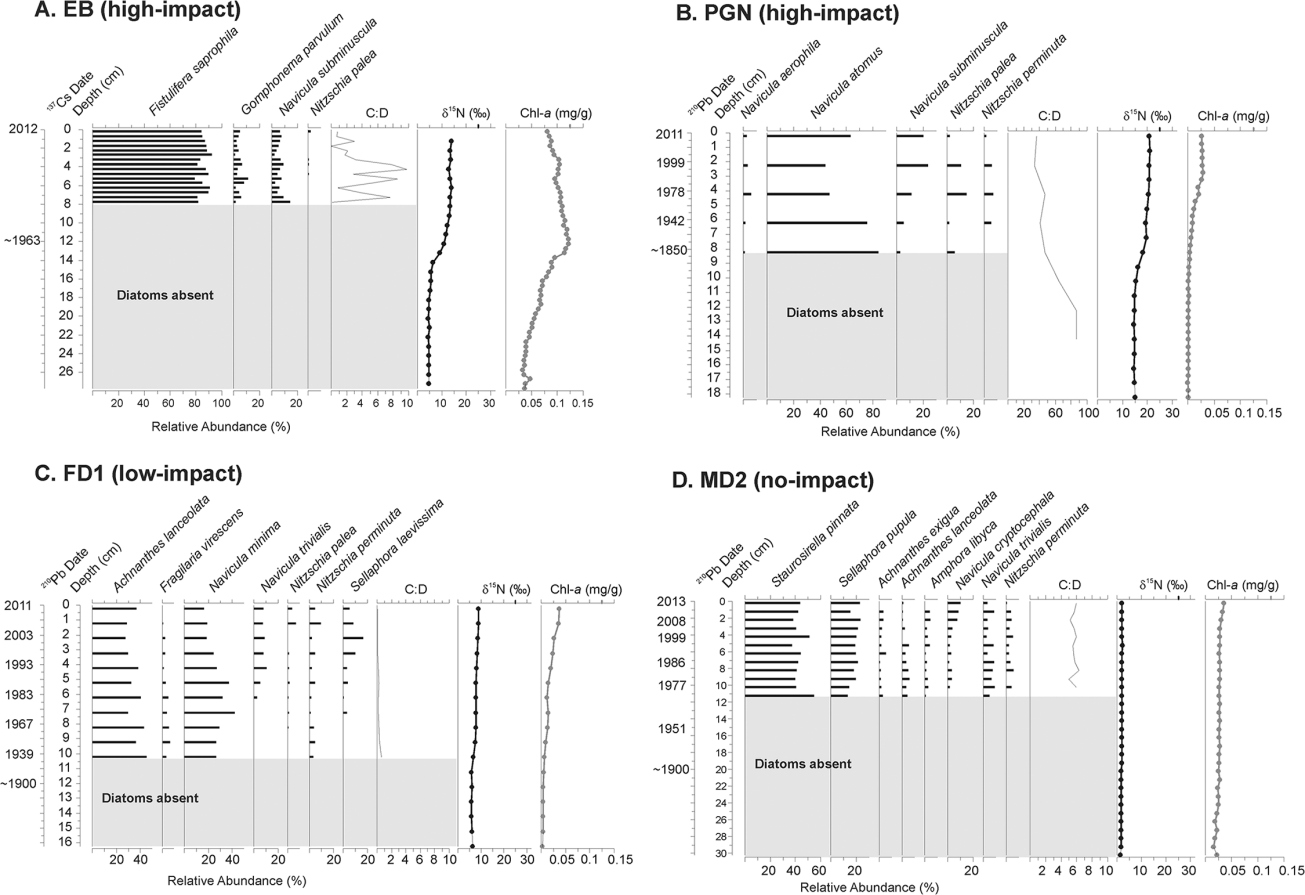
Subfossil diatom assemblages and geochemical tracers from sediment cores. Data are shown for the high-impact ponds on (A) East Brother Island (EB) and (B) Pigeon Island (PGN), as well as from the reference sites on (C) False Duck Island (FD1) and (D) Main Duck Island (MD2). The cyst to diatom ratio (C:D), sedimentary δ^15^N (‰) and spectrally-inferred sedimentary chlorophyll-*a* (chl-*a*) concentrations (mg/g) are given to the right of each stratigraphy. All profiles are presented on the same scales, except the C:D ratio for PGN. Grey boxes at the bottom of each core indicate sediment intervals in which diatom remains were too sparse to be enumerated.

The ^210^Pb radioactivity profiles for the sediment cores collected from PGN, FD1, and MD2 showed decay with time, allowing for the development of a ^210^Pb-based chronology (S1 Fig). In the EB core, the ^210^Pb radioactivity profile indicated mixing from the surface to a depth of ~12 cm (S1 Fig). The intrinsic time resolution, or estimation of the span of time that mixing has affected [33], is ~60 years for the EB core. The peak in ^137^Cs radioactivity (indicating the height in atomic bomb testing circa 1963) occurred at ~13.5 cm depth, which is below the mixed depth of 12 cm, and was therefore reported (Fig 3A). However, it should be noted that there is a discrepancy of ~10 years between the intrinsic time resolution of the mixed layer (60 years) and ^137^Cs peak (~1963), and thus this independent dating marker should be used as an approximate guideline for the mid-19^th^ century only.

### Sedimentary δ^15^N and spectrally-inferred chlorophyll-a

Similar to large differences in water chemistry between bird-affected sites and bird-free sites, sedimentary δ^15^N is higher in EB and PGN (high-impact ponds) than in FD1 and MD2 (low- and no-impact ponds, respectively) throughout the sediment records (Fig 3). Increases in sedimentary δ^15^N occurred in EB (high-impact) from ~5 to 13.5‰ between 14 and 12 cm depth (~ mid-19^th^ century). The sedimentary δ^15^N profile in PGN (high-impact) increased around 11 cm (pre-1850) from a minimum of ~15‰ to a maximum of ~20‰. Even the low-impact pond, FD1, experienced a small increase in δ^15^N from ~6 to 9‰ gradually over the entire sediment record (beginning before ~1900). However, sedimentary δ^15^N in MD2 (no-impact) remained low at ~1–2‰ for the duration of the record.

Similarly timed changes to those recorded in our δ^15^N data occurred in chl-*a* in EB, PGN, and FD1 (Fig 3), with values in EB exceeding those of PGN and FD1. Chl-*a* in EB (high-impact) shifted from 0.05 mg/g to 0.1 mg/g around 12–14 cm (~ mid-19^th^ century). Chl-*a* concentrations were at or below detection limits (~0.01 mg/g) at 14 cm or deeper in PGN (high-impact), and increased notably at ~5 cm (~1960), reaching a maximum of ~0.025 mg/g at the surface. In contrast, chl-*a* in FD1 (low-impact) remain stable at >0.01 mg/g from the bottom of the core until 12 cm (~1900), and then gradually increased to 0.04 mg/g at the surface. Chl-*a* in MD2 (no-impact) stayed approximately constant at 0.25 mg/g with a slight increase to 0.36 mg/g at the surface.

### Subfossil diatom assemblages

Common diatom taxa (greater than 5% relative abundance) were plotted for all cores, and large floristic differences were noted between the high-impact sites and reference sites (Fig 3). In all study ponds, diatom remains were too sparse to enumerate in the lower sections of the cores (with signs of dissolution), although siliceous chrysophyte cysts were well-preserved throughout the sedimentary record. The diatom assemblages recovered from EB (high-impact) were dominated by *Fistulifera saprophila* (>80%) and the C:D fluctuated between 0–8 throughout. The diatom assemblage in PGN (high-impact) consisted primarily of *Navicula atomus* (~60%), as well as lower abundances of *Navicula aerophila*, *Navicula subminiscula*, *Nitzschia palea*, and *Nitzschia perminuta* (Fig 3B). A decrease in *N*. *atomus* was concurrent with increases in *N*. *subminiscula*, *N*. *palea*, and *N*. *perminuta* between 8 and 2 cm. The C:D in PGN decreased from ~90 at 14 cm to ~40 at the surface. In the FD1 core (low-impact), the diatom assemblage was comprised of primarily *Achnanthes lanceolata* (~20–40%) and *Navicula minima* (~20–40%), with subtle shifts occurring between these benthic taxa and some less dominant species (Fig 3C). The C:D in FD1 remained stable at <1 throughout the core. The MD2 (no-impact) sediment core was dominated by *Staurosirella pinnata* (~50%) and *Sellaphora pupula* (~20%) throughout, with low abundances of other benthic taxa (Fig 3D). Again, some subtle shifts between the benthic taxa of MD2 are noted, as with FD1. The C:D for the MD2 core remained stable near values of ~6 throughout.

## Discussion

Our limnological and paleolimnological comparisons of ponds differentially affected by double-crested cormorants and ring-billed gulls clearly document the impacts of dense waterbird colonies on local ecosystems. Furthermore, geochemical and biological proxies preserved in dated sediment cores highlight the potential for using paleolimnology to qualitatively extend estimates of cormorant occupation and influence into the past where monitoring records are absent. Below we summarize these findings.

### High-impact sites (East Brother Island and Pigeon Island)

Our water chemistry analyses demonstrate the fertilizing effects of cormorant and gull guano. The elevated specific conductivity and nutrient (TP-u and TN-u) concentrations in the cormorant-affected EB site compared to reference sites (Fig 2) reflect the large amounts of high ionic and nutrient content waste deposited by the >3000 cormorants that nest in the area from April to September. Even though detailed water chemistry was unavailable for PGN (because of low water depths), the extremely elevated specific conductivity (1650 μS/cm) at this site, surpassing that of EB (~800 μS/cm) by more than double (Fig 2), clearly reflects the influence of ornithogenic waste inputs. Indeed, this is not a surprising finding considering the enrichment of nitrates, phosphates, and potassium ions in soils impacted by cormorants has previously been documented in the Gulf of Mexico [34] and by cormorants (*Phalacrocorax carbo sinensis*) in Poland [35]. Furthermore, limnological surveys in shallow Arctic ponds affected by seabirds record similar water chemistry changes [36]. The effect that pond size has on water chemistry measurements should also be noted, as PGN is much smaller than EB and therefore more susceptible to evaporative concentration of solutes.

While the limnological effects of cormorant waste on modern water chemistry is undeniable, only the sediment core data can assess the pre-monitoring history of cormorants on these islands. With a multi-proxy paleolimnological approach, we show the strong influences that cormorants and gulls have on sedimentary geochemical profiles, which can be used to track past populations, as well as the aquatic ecological changes that accompany their arrival. The δ^15^N value in EB from 28–12 cm is approximately 5‰, which is close to the background values of Lake Ontario sediments before human impact (~4‰, [27]). The sharp rise in sedimentary δ^15^N to ~13.5‰ around 13 cm is close to values recorded in cormorant tissues (15‰, [24]). The increase in δ^15^N occurs synchronously with a rise in chl-*a* around the mid-1960s (13 cm depth), suggesting cormorants arrived on the island and, as a result, nutrient-rich wastes increased the primary production of the pond. Although these two independent proxies could demarcate an easily interpretable timeline for cormorant arrival, historical records document that cormorants did not arrive to East Brother Island until 2001 (S1 Table), postdating the change in our proxies by four decades according to our ^137^Cs independent dating marker. This discrepancy in timing is possibly due to ornithogenic bioturbation of the sediments (as reflected in the ^210^Pb radioactivity profile, S1 Fig) that likely occurred when cormorants first appeared in large numbers on the island. Cormorants (especially the young) have been observed wading into the pond on East Brother Island, however mixing of the sediments could also be due to strong winds and storm events, as the pond becomes shallower toward the end of the summer due to evaporation. Nonetheless, the EB core clearly reflects the influence of cormorants as the diatom assemblage is dominated (>80%) by *Fistulifera saprophila*, which can tolerate hyper-eutrophic and highly polluted waters [37, 38]. The C:D of EB also tracks the eutrophic nature of the pond [31], as it remains low (but fluctuating) in the top 8 cm of the core. Overall, the paleolimnological analysis of EB demonstrates that cormorants not only leave strong geochemical signatures in sediments that are useful for qualitative observations of past occupation, but they also drastically alter the ecology of the pond in agreement with the vast enrichment of nutrients and ions in the water.

In the PGN core, sedimentary δ^15^N values of 15‰ in pre-1850s sediment indicate that birds have been nesting on Pigeon Island for the period encompassed by the core. As ring-billed gulls were common in this area in the 19^th^ century [39] and the island was dominated by this species up until recently [17], it is possible that Pigeon Island has been a long used habitat for ring-billed gull colonies. Furthermore, cormorants were common in North America at this time [40], and may have used Pigeon Island for nesting, though there are no historical documents to confirm this possibility. In PGN, the gradual increase in sedimentary δ^15^N that began around 11 cm (~1850) indicates an increase in influence of higher trophic levels, which could be attributed to the building of a lighthouse and permanent settlement of people on Pigeon Island in 1870 (contributing human wastes to the landscape), as well as the increasing populations of ring-billed gulls that occurred later in the 1930s and 1940s [16]. This increase to 20‰ is higher than even those δ^15^N values recorded in cormorant tissues [24], and further δ^15^N enrichment by denitrification and ammonification is likely to be occurring. In particular, volatilization of ammonia is expected at pH 9, and this might elevate δ^15^N levels in the sediment because ^14^N is preferentially evaporated [41]. Denitrification occurs generally under anaerobic conditions resulting in enhanced δ^15^N in sediments. This could also be further exacerbated by recent climate warming, which has been documented to affect many freshwaters in the Northern Hemisphere [42]. Although the δ^15^N signal could be altered following its release into these ponds, these effects should collectively serve to enrich δ^15^N under conditions of high ornithogenic nutrient inputs. It is therefore not surprising that the arrival of cormorants on the island in the 1960s [17] is not recorded by any further increases in δ^15^N, as the already elevated background values of δ^15^N (~15‰) early in the sediment record is equal to that of cormorant tissues. Nonetheless, the increase in sedimentary chl-*a* circa 1960 (5 cm depth) indicates rising primary production, likely tracking the arrival of cormorants (as documented in [17]) and their large nutrient inputs, as well as the continuing increase in ring-billed gulls [15]. Here, the use of multiple proxies facilitates the interpretation of possible historical bird occupation.

The diatom assemblage of PGN (high-impact) reflects the current eutrophic nature of the pond, and possibly the arrival of cormorants to the island. Here the diatom assemblage is dominated by *Navicula atomus*, a eutrophic and aerophilic species [43], reflecting the nutrient-rich and ephemeral nature of this pond. Changes in diatom relative abundance also appear to track cormorant influence with the presence of *Navicula subminuscula* and *Nitzschia palea* at relative abundances of 10–20%, both of which are tolerant of high nutrients and organic pollution [43, 44] and begin increasing in relative abundance in synchrony with chl-*a* concentrations around 1960 (5 cm depth) in PGN. This suggests that the arrival of cormorants increased primary production and led to a shift to more eutrophic diatom species. The C:D also tracks the eutrophication of PGN, as it decreases from the bottom of the sediment record to the surface, indicating declining proportions of oligotrophic chrysophytes compared to diatoms as the pond became more productive [31]. However, it should be noted that the C:D of PGN is much higher than all other study ponds regardless of bird impacts, which could be related to the extremely ephemeral nature of PGN. Similar to the paleolimnological evidence from EB, the sediment record from PGN contains distinct signals of cormorant influence that are consistent with historical records and strengthened when using a multi-proxy approach.

### Reference sites (False Duck Island and Main Duck Island)

Although both of our reference sites are nutrient-rich temperate ponds adjoined to marshes, they do not exhibit the elevated nutrient and ionic concentrations of our bird-affected sites (Table 1, Fig 2). This is evident in the specific conductivity values that range from ~200–400 μS/cm in the reference ponds MD2 and FD1 (which are close to 40-year averages from Lake Ontario of ~300 μS/cm [45]), compared to high-impact ponds EB and PGN that have elevated values of 800 and 1650 μS/cm, respectively (Fig 2A). The drastic difference in water chemistry between impacted and reference ponds is particularly evident in nutrient measures that show approximately doubled concentrations of nitrogen in ponds receiving cormorant wastes, and even more with phosphorus, which is 40 times greater in EB (high-impact) compared to MD2 (no-impact). Because all of our islands are located in similar geographic regions, it is reasonable to conclude that strong variations in water chemistry are due to the presence of dense cormorant colonies at our high-impact sites, which not only have direct effects on water chemistry through waste inputs, but also very likely indirect or latent effects due to drastic alterations to the catchments that are intimately linked to the ponds.

We also analyzed sediment cores from ponds with little-to-no bird activity to compare with the paleolimnological history of our bird-affected sites. In the low-impact pond (FD1), a slow subtle rise in δ^15^N and chl-*a* occurred around 1940 until the present (Fig 3B). This change is minor compared to the dramatic increases at our high-impact sites and is likely due to the presence of other nesting birds such as ring-billed gulls, which returned to this area of Lake Ontario in the 1930s and subsequently experienced large population increases after World War II as a result of adapting to urban resources like landfills [16, 25]. Because FD1 is part of a marsh, it does not have large colonies of cormorants or gulls in its immediate catchment (because they nest on solid and open ground), and therefore records only subtle influences in its sediment record. Moreover, the diatom assemblage of FD1 (low-impact) was dominated by benthic species that do not indicate large nutrient additions (Fig 3). The low C:D throughout, however, does reflect the productive nature of FD1, as well as a lack of the open-water habitat in which many chrysophytes thrive (as FD1 is a marsh) [31].

As expected, the sediments from our no-impact site (MD2) recorded unchanging low values of sedimentary δ^15^N that reflect the absence of waterbirds at this site over the time period recorded in the sediments. The δ^15^N values of MD2 (1–2 ‰) are even lower than those of pre-settlement Lake Ontario sediments (~4‰, [27]), reflecting the variable nature of background sedimentary δ^15^N, which may differ depending on catchment inputs and post-depositional processes [41]. Only slight increases in chl-*a* concentrations were noted near the surface of the core (~2008), which can likely be attributed to recent climate warming that has been linked to increases in primary production in temperate regions [46]. Since this increase in chl-*a* is much smaller than those recorded in the impacted ponds (Fig 3), it is likely that the large increases in primary production at the impacted sites are due to cormorant waste inputs and not solely an external factor such as climate change. Similarly, the diatom assemblage of MD2 (no-impact) is characterized by benthic species that do not indicate high levels of nutrient inputs, but are still tolerant of eutrophic conditions, which appear to naturally exist in the productive marsh habitat of MD2. Similarly to the C:D of the FD1 sediment record, MD2 had stable low values for C:D that indicate its eutrophic water chemistry.

## Conclusions

Current limnological sampling and several palaeolimnological proxies at our sites all record the influence of ornithogenic inputs in a manner *a priori* predicted based on previous studies of biovectors on freshwaters [1]. Although isotope-based paleolimnological studies of bird colonies have thus far been largely limited to the remote Arctic, we show the potential for using these methods in temperate regions, particularly the North American Great Lakes, which are surrounded by dense urban centres. The large differences in production-related variables between sites with extensive versus minimal bird activity further confirms the efficacy of this approach in tracking waterbird impacts on freshwaters. Of considerable interest to Great Lakes managers is the fact that the high density of cormorants on East Brother Island is likely unprecedented for at least the past ~100 years, but not necessarily so at Pigeon Island over the past ~150 years, suggesting that different management strategies may be appropriate for different sites. This knowledge is particularly salient because cormorants are susceptible to population management for socio-economic reasons (e.g. reducing competition with sports fisheries) and thus consideration of past population levels is key [11]. However, it should be noted that double-crested cormorant colonies may move to different islands over time. With this in mind, paleolimnology can be used to determine if and when cormorants have used specific islands of economic or cultural importance as nesting sites in order to make historically-informed management decisions that are not entirely based on human interests. Furthermore, ongoing developments in the field of paleolimnology, such as the use of taxon-specific stanol and sterol biomarkers [47], may eventually be used to distinguish different waterbird species, which would be particularly useful for separating inputs from cormorants versus ring-billed gulls in the sedimentary record, as they often share nesting sites.

Previous research on the role of Arctic seabirds as biovectors of contaminants [3] has demonstrated that birds which feed at high trophic positions and nest in large colonies, have the potential to deposit contaminants including persistent organic pollutants and heavy metals, in concentrations far beyond that of atmospheric deposition. Similar paleolimnological work for cormorant-affected sites could have strong implications for environmental monitoring in the Great Lakes, as this species has the potential to biomagnify contaminants from the Great Lakes on the numerous islands that act as nesting grounds in the summer. Our ongoing research will continue to expand our regional survey of nesting islands, and thus provide a broader historical perspective of past waterbird populations in this area of concern.

## Supporting Information

S1 Fig Measurements of ^210^Pb, ^137^Cs, and ^214^Bi from the sediment cores of the study ponds. Data are shown for the high-impact ponds on (A) East Brother Island (EB) and (B) Pigeon Island, as well as from the reference sites on (C) False Duck Island (FD1) and (D) Main Duck Island (MD2). Error bars represent modeled error from the ScienTissiME program for Matlab (Barry’s Bay, ON, Canada).
(DOCX)

S1 Table Cormorant census data from D.V.C. Weseloh for East Brother Island. No cormorants were noted on East Brother Island prior to 2001 and none prior to 1986 on False Duck Island.
(DOCX)

S2 Table Selected water chemistry variables for the impacted pond on East Brother Island (EB). Samples were collected in the summer (June 24^th^) and fall (September 11^th^) of 2013. Abbreviations are as follows: dissolved organic carbon (DOC), dissolved inorganic carbon (DIC), particulate organic carbon (POC), particulate organic nitrogen (PON), total nitrogen (TN), and total phosphorus (TP).
(DOCX)

S3 Table Selected water chemistry variables for study ponds. Included are the high-impact pond on East Brother Island (EB) and the low-impact pond on False Duck Island (FD1) from samples taken on May 28^th^ 2012. Samples were also collected from the no-impact pond on Main Duck Island (MD2) on September 11^th^ 2013. Major ions concentrations are given as unfiltered values for EB and FD1, and as filtered values for MD2. Only field measures of pH and specific conductivity (μS/cm) were collected from the high-impact pond on Pigeon Island (PGN) on June 16^th^ 2011. Abbreviations are as follows: dissolved organic carbon (DOC), dissolved inorganic carbon (DIC), particulate organic carbon (POC), particulate organic nitrogen (PON), total nitrogen (TN), and total phosphorus (TP).
(DOCX)

## References

[1] Blais JM, Macdonald RW, Mackey D, Webster E, Harvey C, Smol JP. Biologically mediated transport of contaminants to aquatic systems. Environ Sci Technol. 2007;41: 1075–1084. 10.1021/es061314a 17593703

[2] Krümmel E, Macdonald RW, Kimpe LE, Gregory-Eaves I, Demers M, Smol J, et al Delivery of pollutants by spawning salmon. Nature. 2003;425: 255–256. 10.1038/425255a 13679906

[3] Blais JM, Kimpe LE, McMahon D, Keatley BE, Mallory ML, Douglas MSV, et al Arctic seabirds transport marine-derived contaminants. Science. 2005;309: 445 10.1126/science.1112658 16020729

[4] Ellis JC, Fariña JM, Witman JD. Nutrient transfer from sea to land: the case of gulls and cormorants in the Gulf of Maine. J Anim Ecol. 2006;75: 565–574. 10.1111/j.1365-2656.2006.01077.x 16638009

[5] Boutin C, Dobbie T, Carpenter D, Herbert CE. Effects of double-crested cormorants (*Phalacrocorax auritus* Less.) on island vegetation, seedbank, and soil chemistry: evaluating island restoration potential. Restor Ecol. 2011;19: 720–727. 10.1111/j.1526-100X.2010.00769.x

[6] Glahn JF, Stickley AR Jr.. Wintering double-crested cormorants in the delta region of Mississippi: Population levels and their impact on the catfish industry. Waterbirds. 1995;18 (Special Issue 1): 137–142. 10.2307/1521533

[7] Lantry BF, Eckert TH, Schneider CP, Chrisman JR. The relationship between the abundance of smallmouth bass and double-crested cormorants in the eastern basin of Lake Ontario. J Great Lakes Res. 2002;28: 193–201. 10.1016/S0380-1330(02)70576-5

[8] Cuthbert FJ, Wires LR, McKearnan JE. Potential impacts of nesting double-crested cormorants on great blue herons and black-crowned night-herons in the U.S. Great Lakes Region. J Great Lakes Res. 2002;28: 145–154. 10.1016/S0380-1330(02)70572-8

[9] Doucette JL, Wissel B, Somers CM. Cormorant–fisheries conflicts: Stable isotopes reveal a consistent niche for avian piscivores in diverse food webs. Ecol Appl. 2011;21: 2987–3001. 10.1890/10-2384.1

[10] Weseloh DVC, Pekarik C, Havelka T, Barrett G, Reid J. Population trends and colony locations of double-crested cormorants in the Canadian Great Lakes and immediately adjacent areas, 1990–2000: A manager’s guide. J Great Lakes Res. 2002;28: 125–144. 10.1016/S0380-1330(02)70571-6

[11] Wires LR, Cuthbert FJ. Historic populations of the double-crested cormorant (*Phalacrocorax auritus*): Implications for conservation and management in the 21^st^ century. Waterbirds. 2006;29: 9–37. 10.2307/4132602

[12] Weseloh DVC, Ewins PJ, Struger J, Mineau P, Bishop CA, Postupalsky S, et al Double-crested cormorants of the Great Lakes: Changes in population size, breeding distribution and reproductive output between 1913 and 1991. Waterbirds. 1995;18 (Special Issue 1): 48–59. 10.2307/1521523

[13] Weseloh DVC, Cuthbert FJ, King T. Introduction: Double-crested cormorants of the Great Lakes–St. Lawrence River Basin: Recent studies, movements and responses to management actions. Waterbirds. 2012;35 (Special Issue 1): 1–3. 10.1675/063.035.sp101

[14] Blokpoel H, Tessier GD. Distribution and abundance of colonial waterbirds nesting in the Canadian portions of the lower Great Lakes system in 1990. Toronto (ON): Canadian Wildlife Service, Ontario Region; 1991. Technical Report Series No. 117. Sponsored by Environment Canada.

[15] Morris RD, Weseloh DV, Wires LR, Pekarik C, Cuthburt FJ, Moore DJ. Population trends of ring-billed gulls breeding on the North American Great Lakes, 1976 to 2009. Waterbirds. 2011;34: 202–212. 10.1675/063.034.0209

[16] Ludwig JP. Recent changes in the ring-billed gull population and biology in the Laurentian Great Lakes. Auk. 1975;91: 575–594. 10.2307/4084477

[17] Weir RD. Birds of the Kingston Region. 2nd ed. Kingston: Kingston Field Naturalists; 2008.

[18] Environment Canada Manual of Analytical Methods Major Ions and nutrients. vol 1 Burlington: National Laboratory for Environmental Testing, Canadian Centre for Inland Waters; 1994.

[19] Environment Canada Manual of Analytical Methods Trace Metals. vol 2 Burlington: National Laboratory for Environmental Testing, Canadian Centre for Inland Waters; 1994.

[20] Glew JR. A new trigger mechanism for sediment samplers. J Paleolimnol. 1989;2: 241–243. 10.1007/BF00195474

[21] Glew JR. A portable extruding device for close interval sectioning of unconsolidated core samples. J Paleolimnol. 1988;1: 235–239. 10.1007/BF00177769

[22] Appleby PG. Chronostratigraphic techniques in recent sediments In: Last WM, Smol JP, editors. Tracking environmental change using lake sediments. vol 1: Basin analysis, coring and chronological techniques Dordrecht: Kluwer Academic Publishers; 2001 pp. 172–203.

[23] Yamamuro M, Kayanne H. Rapid direct determination of organic-carbon and nitrogen in carbonate-bearing sediments with a Yanaco mt-5 CHN analyzer. Limnol Oceanogr. 1995;40: 1001–1005. 10.4319/lo.1995.40.5.1001

[24] Robinson SA, Forbes MR, Hebert CE. Parasitism, mercury contamination, and stable isotopes in fish-eating double-crested cormorants: no support for the co-ingestion hypothesis. Can J Zool. 2009;87: 740–474. 10.1139/Z09-062

[25] Belant JL, Ickes SK, Seamans TW. Importance of landfills to urban-nesting herring and ring-billed gulls. Landsc Urban Plan. 1998;43: 11–19. 10.1016/S0169-2046(98)00100-5

[26] Caron-Beaudoin É, Gentes M-L, Patenaude M, Hélie J-F, Giroux J-F, Verreault J. Combined usage of stable isotopes and GPS-based telemetry to understand the feeding ecology of an omnivorous bird, the Ring-billed Gull (Larus delawarensis). Can J Zool. 2013;91: 698–697. 10.1139/cjz-2013-0008

[27] Hodell DA, Schelske CL. Production, sedimentation, and isotopic composition of organic matter in Lake Ontario. Limnol Oceanogr. 1998;43: 200–214. 10.4319/lo.1998.43.2.020

[28] Michelutti N, Blais JM, Cumming BF, Paterson AM, Rühland K, Wolfe AP, et al Do spectrally-inferred determinations of chlorophyll a reflect trends in lake trophic status? J Paleolimnol. 2010;43: 205–217. 10.1007/s10933-009-9325-8

[29] Battarbee RW, Jones VG, Flower RJ, Cameron NG, Bennion H, Carvalho L, et al Diatoms In: Last WM, Smol JP, editors. Tracking environmental change using lake sediments. vol. 3: Terrestrial, Algal, and Siliceous Indicators Dordrecht: Kluwer Academic Publishers; 2001 pp.155–202.

[30] Krammer K, Lange-Bertalot H. Bacillariophyceae. Stuttgart: Gustav Fisher Verkag; 1986–1991.

[31] Smol JP. The ratio of diatom frustules to chrysophycean statospores: a useful paleolimnological index. Hydrobiologia. 1985;123: 199–208. 10.1007/BF00034378

[32] Smol JP, Stoermer EF, editors. The Diatoms: Applications for the environmental and earth sciences 2nd ed. Cambridge: Cambridge University Press; 2010.

[33] Eisenreich SJ, Capel PD, Robbins JA, Bourbonniere R. Accumulation and diagenesis of chlorinated hydrocarbons in lacustrine sediments. Environ Sci Technol. 1989;23: 1116–1126. 10.1021/es00067a009

[34] Wait DA, Aubrey DP, Anderson WB. Seabird guano influences on desert islands: soil chemistry and herbaceous species richness and productivity. J Arid Environ. 2005;60: 681–695. 10.1016/j.jaridenv.2004.07.001

[35] Ligeza S, Smal H. Accumulation of nutrients in soils affected by perennial colonies of piscivorous birds with reference to biogeochemical cycles of elements. Chemosphere. 2003;52: 595–602. 10.1016/S0045-6535(03)00241-8 12738297

[36] Michelutti N, Keatley BE, Brimble S, Blais JM, Liu H, Douglas MSV, et al Seabird-driven shifts in Arctic pond ecosystems. Proc Roy Soc B. 2009;276: 591–596. 10.1098/rspb.2008.1103 PMC2664339 18945662

[37] Spaulding S, Edlund M. Fistulifera; 2008. Database: Diatoms of the United States [Internet]. Available: http://westerndiatoms.colorado.edu/taxa/genus/Fistulifera.

[38] Stewart EM, McIver RM, Michelutti N, Douglas MSV, Smol JP. Assessing the efficacy of chironomid and diatom assemblages in tracking eutrophication in High Arctic sewage ponds. Hydrobiologia 2014;721: 251–268. 10.1007/s10750-013-1667-6

[39] Audubon JJ. The Birds of America. vol. 7 New York: J.J. Audubon; 1844.

[40] Audubon JJ. The Birds of America. vol. 6 New York: J.J. Audubon; 1843.

[41] Kendall C, McDonnell JJ. Isotope Tracers in Catchment Hydrology. New York: Elsevier Science; 1998.

[42] Williamson CE, Saros JE, Vincent W, Smol JP. Lakes and reservoirs as sentinels, integrators, and regulators of climate change. Limnol Oceanogr. 2009;54: 2273–2282. 10.4319/lo.2009.54.6_part_2.2273

[43] Van Dam H, Mertens A, Sinkeldam J. A coded checklist and ecological indicator values of freshwater diatoms from The Netherlands. Neth J Aquat Ecol. 1994;28: 117–133. 10.1007/BF02334251

[44] Rott E, Duthie HC, Pipp E. Monitoring organic pollution and eutrophication in the Grand River, Ontario, by means of diatoms. Can J Fish Aquat Sci. 1998;5: 1443–1453. 10.1139/f98-038

[45] Dove A. Long-term trends in major ions and nutrients in Lake Ontario. Aquat Eco Sys Health Manag. 2009;12: 281–295. 10.1080/14634980903136388

[46] Hyatt CV. A diatom-based paleolimnological investigation of historical water-quality and ecological changes in the Lake of the Woods, Ontario. M.Sc. Thesis. Queen’s University. 2010. Available: https://qshare.queensu.ca.

[47] Korosi JB, Cheng W, Blais JM. Organic Pollutants in Sediment Core Archives In: Blais JM, Rosen MR, Smol JP, editors. Developments in Paleoenvironmental Research. vol 18: Environmental Contaminants: Using Natural Archives to Track Sources and Long-Term Trends of Pollution Dordrecht: Springer; 2015 pp. 161–186. 10.1007/978-94-017-9541-8_8

