# Supplementary material for: Tracking the History and Ecological Changes of Rising Double-Crested Cormorant Populations Using Pond Sediments from Islands in Eastern Lake Ontario
Source: PLoS One. 2015 Jul 27;10(7):e0134167. doi: 10.1371/journal.pone.0134167 (PMC4516326; doi:10.1371/journal.pone.0134167)
Supplement: S1 Fig — Data are shown for the high-impact ponds on (A) East Brother Island (EB) and (B) Pigeon Island, as well as from the reference sites on (C) False Duck Island (FD1) and (D) Main Duck Island (MD2). Error bars represent modeled error from the ScienTissiME program for Matlab (Barry’s Bay, ON, Canada). (DOCX) [file pone.0134167.s001.docx]

**
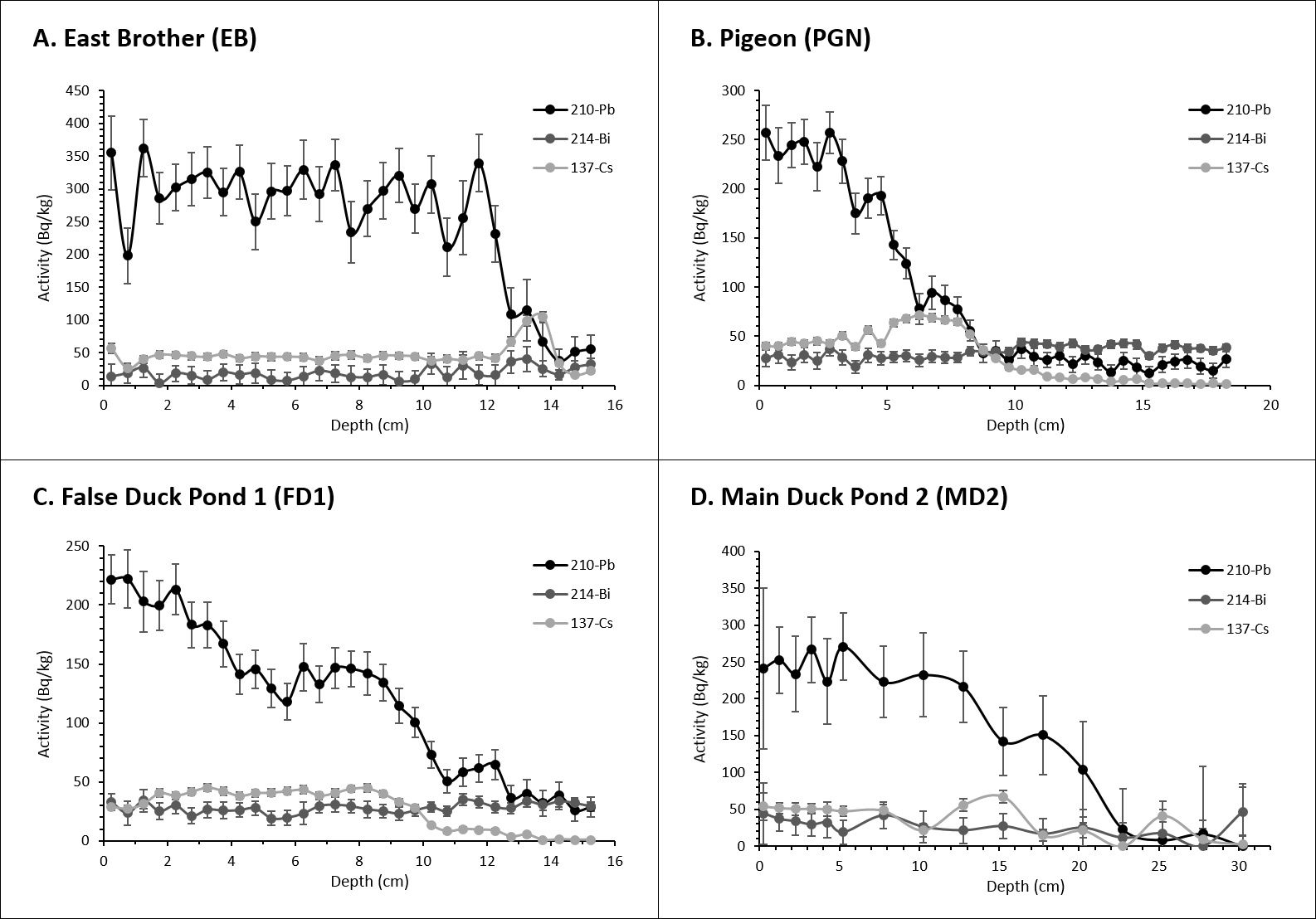
**

**S1 Figure.** **Radioactivities of ^210^Pb, ^137^Cs, and ^214^Bi measured in the sediment cores.** Data is shown for the high-impact ponds on A. East Brother Island (EB) and B. Pigeon Island, as well as from the reference sites on C. False Duck Island (FD1) and D. Main Duck Island (MD2). Error bars represent standard deviation.
