# Supplementary material for: Tracking the History and Ecological Changes of Rising Double-Crested Cormorant Populations Using Pond Sediments from Islands in Eastern Lake Ontario
Source: PLoS One. 2015 Jul 27;10(7):e0134167. doi: 10.1371/journal.pone.0134167 (PMC4516326; doi:10.1371/journal.pone.0134167)
Supplement: S1 Table — No cormorants were noted on East Brother Island prior to 2001 and none prior to 1986 on False Duck Island. (DOCX) [file pone.0134167.s002.docx]

**S1 Table. Cormorant census data from D.V.C. Weseloh for East Brother Island.** No cormorants were noted on East Brother Island prior to 2001 and none prior to 1986 on False Duck Island.

| **Year** | **# of nests** |
| --- | --- |
| 2001 | 197 |
| 2002 | 648 |
| 2003 | 1122 |
| 2004 | 1836 |
| 2005 | 1150 |
| 2006 | 1161 |
| 2007 | 1200 |
| 2008 | 1305 |
| 2009 | 1632 |
| 2010 | 1062 |
| 2011 | 1491 |
| 2012 | 1452 |
