# Supplementary material for: Tracking the History and Ecological Changes of Rising Double-Crested Cormorant Populations Using Pond Sediments from Islands in Eastern Lake Ontario
Source: PLoS One. 2015 Jul 27;10(7):e0134167. doi: 10.1371/journal.pone.0134167 (PMC4516326; doi:10.1371/journal.pone.0134167)
Supplement: S2 Table — Samples were collected in the summer (June 24th) and fall (September 11th) of 2013. Abbreviations are as follows: dissolved organic carbon (DOC), dissolved inorganic carbon (DIC), particulate organic carbon (POC), particulate organic nitrogen (PON), total nitrogen (TN), and total phosphorus (TP). (DOCX) [file pone.0134167.s003.docx]

**S2 Table. Selected water chemistry variables for the impacted pond on East Brother Island (EB).** Samples were collected in the summer (June 24^th^) and fall (September 11^th^) of 2013. Acronyms are as follows: dissolved organic carbon (DOC), dissolved inorganic carbon (DIC), particulate organic carbon (POC), particulate organic nitrogen (PON), total nitrogen (TN), and total phosphorus (TP).

| **Variable** | **Summer** | **Fall** |
| --- | --- | --- |
| Ca^2+^ filtered (mg/L) | 110 | 57.8 |
| Mg^2+^ filtered (mg/L) | 11.4 | 7.99 |
| K^+^ filtered (mg/L) | 46.5 | 57.9 |
| Na^+^ filtered (mg/L) | 17.6 | 27.1 |
| Cl^-^ unfiltered (mg/L) | 22 | 34.3 |
| SO_4_^2-^ unfiltered (mg/L) | 76 | 55.1 |
| pH | - | 9 |
| Specific Conductivity (µS/cm) | 910 | 600 |
| Chl-*a* (µg/L) | 1 | 1.4 |
| DOC (mg/L) | 20.5 | 31.6 |
| DIC (mg/L) | 67.8 | 33.5 |
| POC (mg/L) | 5.29 | 1.34 |
| PON (mg/L) | 0.549 | 0.177 |
| TN filtered (mg/L) | 9.97 | 3.11 |
| TP unfiltered (µg/L) | 2970 | 1440 |
