# Supplementary material for: Tracking the History and Ecological Changes of Rising Double-Crested Cormorant Populations Using Pond Sediments from Islands in Eastern Lake Ontario
Source: PLoS One. 2015 Jul 27;10(7):e0134167. doi: 10.1371/journal.pone.0134167 (PMC4516326; doi:10.1371/journal.pone.0134167)
Supplement: S3 Table — Included are the high-impact pond on East Brother Island (EB) and the low-impact pond on False Duck Island (FD1) from samples taken on May 28th 2012. Samples were also collected from the no-impact pond on Main Duck Island (MD2) on September 11th 2013. Major ions concentrations are given as unfiltered values for EB and FD1, and as filtered values for MD2. Only field measures of pH and specific conductivity (μS/cm) were collected from the high-impact pond on Pigeon Island (PGN) on June 16th 2011. Abbreviations are as follows: dissolved organic carbon (DOC), dissolved inorganic carbon (DIC), particulate organic carbon (POC), particulate organic nitrogen (PON), total nitrogen (TN), and total phosphorus (TP). (DOCX) [file pone.0134167.s004.docx]

**S3 Table. Selected water chemistry variables for study ponds.** Included are the high-impact pond on East Brother Island (EB) and the low-impact pond on False Duck Island (FD1) from samples taken on May 28^th^ 2012. Samples were also collected from the no-impact pond on Main Duck Island (MD2) on September 11^th^ 2013. Major ions concentrations are given as unfiltered values for EB and FD1, and as filtered values for MD2. Only field measures of pH and specific conductivity (µS/cm) were collected from the high-impact pond on Pigeon Island (PGN) on June 16^th^ 2011. Acronyms are as follows: dissolved organic carbon (DOC), dissolved inorganic carbon (DIC), particulate organic carbon (POC), particulate organic nitrogen (PON), total nitrogen (TN), and total phosphorus (TP).

| **Variable** | **EB** | **PGN** | **FD1** | **MD2** |
| --- | --- | --- | --- | --- |
| (Impact Level) | (High) | (High) | (Low) | (None) |
| Ca^2+^ (mg/L) | 95.7 |  | 81.7 | 48 |
| Mg^2+^ (mg/L) | 10.4 |  | 3.68 | 2.45 |
| K^+^ (mg/L) | 43.1 |  | 3.77 | 0.39 |
| Na^+^ (mg/L) | 18.4 |  | 4.95 | 1.32 |
| Cl^-^ (mg/L) | 24.9 |  | 7.01 | 3.52 |
| SO_4_^2-^ (mg/L) | 51.9 |  | 9.86 | 1.73 |
| pH | 7.6 | 7.0 | 7.3 | 7.6 |
| Specific Conductivity (µS/cm) | 800 | 1650 | 433 | 252 |
| DOC (mg/L) | 19.1 |  | 26.1 | 17.6 |
| DIC (mg/L) | 60.3 |  | 47.3 | 24.3 |
| POC (mg/L) | 1.33 |  | 21.5 | 2.98 |
| PON (mg/L) | 0.203 |  | 3.86 | 0.465 |
| TN unfiltered (mg/L) | 8.79 |  | 7.5 | 1.46 |
| TP unfiltered (µg/L) | 3500 |  | 542 | 86.8 |
